# Probiotic bacteria from 10 different traditional Iranian cheeses: Isolation, characterization, and investigation of probiotic potential

**DOI:** 10.1002/fsn3.2817

**Published:** 2022-03-07

**Authors:** Asma Afshari, Mohammad Hashemi, Milad Tavassoli, Vida Eraghi, Seyyed Mohammad Ali Noori

**Affiliations:** ^1^ 37552 Department of Nutrition Faculty of Medicine Mashhad University of Medical Sciences Mashhad Iran; ^2^ Student Research Committee Department of Food Sciences and Technology Faculty of Nutrition and Food Sciences Tabriz University of Medical Sciences Tabriz Iran; ^3^ Department of Biotechnology Sabzevar Branch Islamic Azad University Sabzevar Iran; ^4^ Nutrition and Metabolic Diseases Research Center Clinical Sciences Research Institute Ahvaz Jundishapur University of Medical Sciences Ahvaz Iran; ^5^ Department of Nutrition School of Allied Medical Sciences Ahvaz Jundishapur University of Medical Sciences Ahvaz Iran

**Keywords:** 16s rRNA gene, cheese, dairy, DNA extraction, lactic acid bacteria, *Lactobacillus*, multiplex PCR, pathogenic bacteria, probiotic

## Abstract

In this study, 10 different traditional Iranian cheeses, which are still consumed by people in rural areas of Iran, were examined to isolate new strains of probiotic bacteria. Isolated bacteria were identified by 16s rRNA gene amplification and subjected to series of in vitro tests to find out their probiotic potential. A total of 2345 colonies were collected and 465 of them were confirmed as lactic acid bacteria (LAB), of which *Lactiplantibacillus plantarum*, *Lactobacillus bulgaricus*, and *Lacticaseibacillus casei* were the top three isolated bacteria. Among the different species of LAB isolated in this study, *Lactip*. *plantarum* was the most isolated species, and seven isolates had the significant criteria for being a probiotic strain than other isolates indicating the most adaptable properties of this species. *Lactiplantibacillus plantarum* was the most resistant bacteria in the bile resistance test and was also the most durable bacteria in gastrointestinal conditions, for example, acidic environment (pH = 2.5) and trypsin. In contrast, *Lacticaseibacillus casei* was the most susceptible bacterial strain. *Lactobacillus rhamnosus* showed the most antibacterial effect against *Staphylococcus aureus*, *Escherichia coli*, and *Pseudomonas aeruginosa*. This study showed that probiotic strains isolated from local cheeses could be considered as suitable biopreservatives and used as specific starter cultures for the production of functional cheeses.

## Graphical abstract

In this study, efforts have been made to isolate new lactic acid bacteria. *Lactiplantibacillus plantarum* was the most isolated species and seven isolates had the major criteria for being a probiotic strain. *Lactip*. *plantarum* was the most resistant bacteria in bile resistance test and was also the most durable bacteria in gastrointestinal conditions, for example, acidic environment (pH = 2.5) and trypsin. This study showed that probiotic strains isolated from local cheeses can be considered as suitable biopreservatives and used as specific starter cultures for the production of functional cheeses.

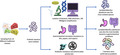

## 1 INTRODUCTION

Many advantages were reported for the consumption of probiotics (Alemohammad et al., 2022; Sharma et al., 2021). Beneficial effects of probiotics depend on their transport and survival through the gastrointestinal tract, which is also affected by initial numbers of probiotics in food, storage condition, food processing, food matrix, etc. (Behare et al., 2021). Among many foods that have been studied for this purpose, cheese is the most interesting case to deliver these bacteria into the intestine (Karimi et al., 2011). This could be associated with the relatively high pH and fat content of cheese which exhibit a protective effect for the survival of probiotics in food and the gastrointestinal tract. Cheese and dairy products have been introduced among the fastest thriving food products in recent years worldwide (Elleuch et al., 2020). Many traditional dairy products and cheeses are produced and consumed in Iran, particularly rural regions. Traditional cheeses such as Lighvan (Ehsani et al., 2018), Koopeh cheese (Ehsani et al., 2018), and Shal fresh cheese could play a role in selecting new potential probiotic bacteria.

Many probiotics have been isolated from cheese, such as lactic acid bacteria (LAB) and *Bifidobacteria* with different strains. Among all LAB, *Lactiplantibacillus plantarum*, *Lactic*. *casei*, *Limosilactobacillus fermentum*, and *Lactobacillus rhamnosus* were more frequent (Ningtyas et al., 2019). LAB have been incorporated with different cheeses such as cheddar cheese, mascarpone cheese, and cottage cheese (Ningtyas et al., 2019). LAB strains are categorized as generally recognized as safe (GRAS) (Tulumoğlu et al., 2014), which makes them suitable for utilization in foods by researchers and industries. Meanwhile, industries seek to produce new probiotic products in response to consumers' high demand.

One of the best approaches to find new probiotic strains is the investigation of traditional foods. These new strains can be used to produce new probiotic foods (Ehsani et al., 2018). To the best of our knowledge, traditional foods which are produced under nonindustrial conditions may contain different and unique bacterial compositions compared to industrial and modern foods (Gupta et al., 2021). Investigation of these traditional foods may lead to the isolation of novel probiotic strains with different properties (Adikari et al., 2021).

Overall, finding novel probiotic strains with high survival rates in food processing stages and high resistance to gastrointestinal juices due to mechanisms such as pH homeostasis, restriction of proton permeation, and enhancement of proton pumps is of great importance to observe their beneficial effects. Finding novel probiotic strains is also essential to provide diversity for probiotic foods and supplements as it has been revealed that diversity of gut microbiota is associated with health (Faintuch & Faintuch, 2019). In this study, we investigated 10 different traditional Iranian cheeses to isolate new strains of LAB. We also analyzed the identified bacteria to find out their resistance and survivability in the rough in vitro conditions of the gastrointestinal tract, as well as their antibacterial activity and their resistance to pathogenic bacteria.

## 2 MATERIALS AND METHODS

### 2.1 Traditional cheese sampling

Ten different traditional cheese samples consisting of Lighvan, Kordi, traditional lactic cheese, Tappe‐Sallam, Onsori, Torkamani type 1, Torkamani type 2, Balouchi, Sistani, and Kermanji were purchased. These traditional cheeses are produced from different milk origins, including sheep, cow, and goat, and each of them has its unique maturation process, such as actual temperatures. The first five samples were collected randomly from some dairy shops located in five districts of Mashhad, which were under the supervision of hygienic centers during the summer of 2018. The second five samples were collected from five different cities of Golestan province, Iran, during the summer of 2019. All samples were provided with three replications, with a total number of 30 samples. The samples were transferred to the Laboratory of Food Hygiene at the Department of Nutrition, Faculty of Medicine, Mashhad University of Medical Sciences. Samples were then immediately subjected to microbiological analysis.

### 2.2 Isolation of bacteria from cheese samples

Isolation of bacteria was carried out according to Hassanzadazar et al. (2017) with slight modifications. Ninety‐milliliter sterile solution of 0.85% sodium chloride (Sigma‐Aldrich) was added to 10 g samples and shaken for 10 min with a shaker (Heidolph) to obtain suspensions. Thirty milliliters of each sample were inoculated into Erlenmeyer flask with 300 ml de Man, Rogosa and Sharp (MRS) broth and incubated at the anaerobic conditions at 37°C for 24 h. Twenty‐five milliliters of the latter was then centrifuged for 20 min at 10,000 g and sedimented mass cells were transferred to 10 ml phosphate buffer solution (pH = 2.5) and incubated at 37°C for 2 h. Subsequently, 0.1 ml sample was spread plated on MRS agar for 24–48 h at 37*°*C anaerobically and random colonies (flat or raised with grayish white color, smooth, rough, or intermediate) were selected for confirmation by some routine tests such as morphological evaluation by a microscope, Gram staining, and catalase test. Bacterial isolates characterization was performed by growth in MRS broth for 5 days at 30°C, 35°C, and 45°C, growth in MRS broth with 3%, 4%, and 6.5% NaCl at 30°C for 2 days, and gas production from glucose, fructose, sorbose, mannose, and xylose (Badis et al., 2004). Confirmed colonies were subcultured three times on MRS media for purification. Confirmed colonies were transferred into brain heart infusion broth (BHI) (Oxoid) supplemented with 15% *(*wt/vol) glycerol and stored at −80°C.

### 2.3 DNA extraction, *16S rRNA* gene amplification by PCR, and phylogenetic inference

DNA extraction was carried out according to the manufacturer's procedure (Qiagen DNA extraction kit) (Gharbi et al., 2019), and the purified DNA was used as a template for the PCR assay. For sequencing assay, the 1532 bp DNA fragment of *16s rRNA* gene was targeted by using 5‐AGAGTTTGATCCTGGCTCAG‐3 and 5‐CTACGGCTACCTTGTTACGA‐3 primers (Denazist Asia) (Hou et al., 2018). The reaction mixture and PCR conditions for both PCRs were performed according to Scarpellini et al. (2002) using only one set of primers as a diagnostic PCR. Amplification reactions were carried out in thermocycler (Techne TC, 3000, England). The DNA was electrophoresed in 1% agarose gel containing ethidium bromide.

The PCR products (*ca*. 1500 bp) were purified and submitted for sequencing at the Macrogen (South Korea). At first, all sequences were trimmed at both ends and compared along with homologous sequences obtained from BLAST analysis (https://blast.ncbi.nlm.nih.gov/Blast.cgi). All of them were aligned using Clustal W and finally were subjected to phylogenetic analysis. Phylogenetic trees were constructed using MEGAX, and unweighted pair group method with arithmetic means (UPGMA) with 1000 replicates bootstrapping was employed. BioEdit Sequence Alignment Editor program (Hall, 1999) was used for trimming and aligning the sequences.

### 2.4 Bile resistance

After the growth of each strain on MRS agar, it was transferred to sterile saline solution (0.85%) to make the 1.0 McFarland suspension. Of the suspension, 10 µl was spotted on the agar plates with 0.3%, 0.5%, 1.0%, and 2.0% (w/v) Ox‐bile (Sigma‐Aldrich) after 30 min, 1, 1.5, and 2 h, respectively. Plates were incubated anaerobically at 37°C and were evaluated after 24 h. Plates with no bacterial colony are considered negative, and the ones with the growth of colonies are considered positive. Plates with the absence of Ox‐bile were considered as control.

### 2.5 Simulation of gastrointestinal juices

The tolerance of bacteria was evaluated by the modified method of Guo et al. (2009). Briefly, the pH of phosphate buffer saline (PBS) (Sigma‐Aldrich) was fixed to 2.5, 3.0, and 4.0 by adding of 1 mol/L HCl and autoclaved at 121°C for 15 min. To simulate the gastric juice, PBS solution was supplemented with pepsin (1:10,000, Sigma Chemical Co.) and sterilized by filtering through 0.22 µm membrane to obtain pepsin solutions with three different pH (2.5, 3.0, and 4.0) and concentration of 3 g/L.

Intestinal juice was provided by supplementation of PBS solution with trypsin (1:250, Sigma Chemical Co.), and pH of buffer solution was adjusted to 8.0 by using one mol/L NaOH. Trypsin solution was filtered through 0.22 µm membrane, and the final concentration reached 1 g/L (Guo et al., 2009).

### 2.6 Determination of transient tolerance

Each strain was incubated in MRS broth at 37°C for 18 h, followed by centrifugation at 2000 *g* for 15 min. Overnight bacterial cell cultures (18 h) were collected, added to sterile normal saline (0.85% NaCl, w/v), and inoculated into simulated gastric juice with different pH (2.5, 3.0, and 4.0) or PBS solution (pH = 2.5, 3.0, and 4.0). Total viable count (TVC) was investigated for both simulated gastric juice and PBS solution at 0, 1, 2, 3, and 4 h at 37°C to assess the resistance to gastric juice.

To simulate the passage of bacteria through the gastrointestinal tract, after 3 h of incubation into the gastric juice (pH = 3.0), 1 ml of each strain was added to 9 ml of simulated intestinal juice with pH = 8.0 and incubated at 37°C. Resistance of bacteria to transit through the small intestine was measured at 37°C by TVC method for 0, 1, 2, 3, 4, 8, and 12 h (Hashemi et al., 2014).

### 2.7 Antibiotic resistance

A single colony of LAB was selected, inoculated into a 10‐ml tube containing Mueller–Hinton broth (Merck; CLSI, 2006), and incubated at 37°C. When turbidity of tube reached 0.5 McFarland, it was streak cultured on a plate containing 90% (w/v) Mueller–Hinton agar and 10% (w/v) MRS dehydrated broth (pH = 6.7). After implantation of the antibiotic disc on the plates, they were incubated at 37°C for 24–48 h.

Samples with a zone diameter of ≤15 mm are considered resistant. All antibiotic susceptibility tests were carried out in triplicate. Antibiotic susceptibility test was performed for amoxicillin (25 µg/disc), ampicillin (10 µg/disc), chloramphenicol (30 µg/disc), erythromycin (15 µg/disc), streptomycin (10 µg/disc), tetracycline (30 µg/disc), vancomycin (30 µg/disc), cefotaxime (30 µg/disc), kanamycin (30 µg/disc), meropenem (10 µg/disc), nalidixic acid (30 µg/disc), gentamycin (10 µg/disc), and ceftazidime (30 µg/disc) that all had been provided from Padtanteb Company. The results showed the percentage of sensitivity to the antibiotics.

### 2.8 Antibacterial activity of probiotic strains

Each strain was cultivated in 30 ml MRS broth at 37°C for 1 day and then centrifuged at 10,000 g for 15 min. A 0.22‐µm filter was used to omit remaining cells from the supernatant. NaOH 1 M was utilized to adjust the pH of 1 ml filtered supernatant (final pH 6.5–7). It was then treated with 0.5 mg/ml catalase to inactivate the hydrogen peroxide of the supernatant. Agar well diffusion method was used to evaluate the antibacterial activity of LAB strains. Five pathogenic bacteria including *Listeria monocytogenes* (ATCC 7644), *Staphylococcus aureus* (PTCC 1431), *Salmonella enterica* subsp. *enterica* serotype Typhimurium (ATCC 14028), *Pseudomonas aeruginosa* (ATCC 9027), and *Escherichia coli* (PTCC 1338) were inoculated in the nutrient broth (Merck) at 37°C for 18 h and cultured on nutrient agar (Merck). Subsequently, with a sterile borer wells were made on the surface of the agar plate (5 mm diameter) and 50 µl of mentioned supernatant from each LAB strain was poured into them. Plates were then held at 4°C for 4 h, followed by aerobic incubation at 37°C for 18 h. The inhibition zone was measured by a digital caliper. An inhibition zone diameter of ≤15 mm and ≥21 mm was considered as resistant and susceptible, respectively. The diameter zone between 15 and 21 mm was recognized as intermediate resistant. All antimicrobial activity tests were performed in triplicate (Ripamonti et al., 2011).

### 2.9 Amplification of *16s rRNA* of LAB isolates and phylogenetic analysis

**FIGURE 1:**
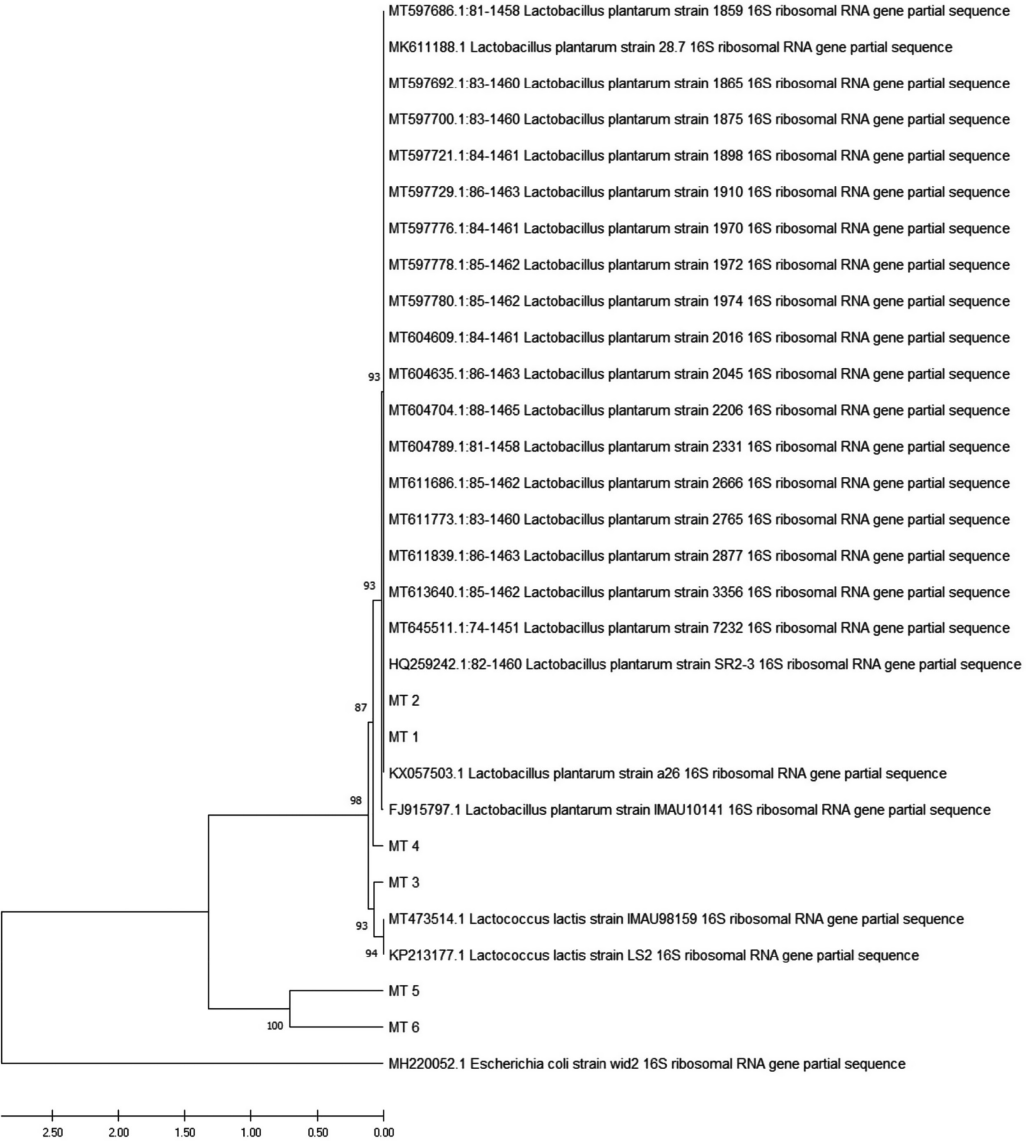
Phylogenetic tree based on showing the relationship between 16S rRNA gene of our sequences (MT1–MT6) along with 23 homologous sequences with the highest similarity. The tree was constructed using MEGAX and UPGMA methods. Branch labels represent the bootstrap values (1000 replicates)

Two trimmed sequences (MT1 and MT2) are available in the GenBank databases under the accession numbers MT000962 and MT000963. Aligned sequences consist of our edited sequences (MT1–MT6) along with 23 homologous sequences that were subjected to phylogenetic analysis. Results of the phylogenetic analysis indicated two main clusters, and our isolates were found in both clusters. Most of our isolates were closely related to strains of *Lactip. plantarum* and one of them (MT3) was clustered along with two isolates of *Lactococcus lactis* strains (Figure 1).

### 2.10 Statistical analysis

Statistical analysis was carried out using SPSS software version 16 (SPSS Inc.) and Microsoft Excel.

## 3 RESULTS AND DISCUSSION

### 3.1 Isolation and identification of probiotic strains from traditional cheeses

A total of 2345 colonies were collected from all kinds of cheese samples, and from each morphological type, at least one colony was selected. Among all colonies, only 489 of them were Gram‐positive and catalase‐negative, and selected as suspected LAB.

**FIGURE 2:**
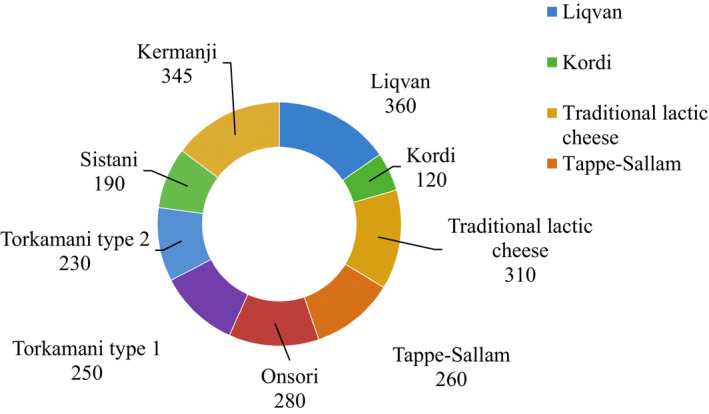
Number of colonies collected from each type of cheese

**FIGURE 3:**
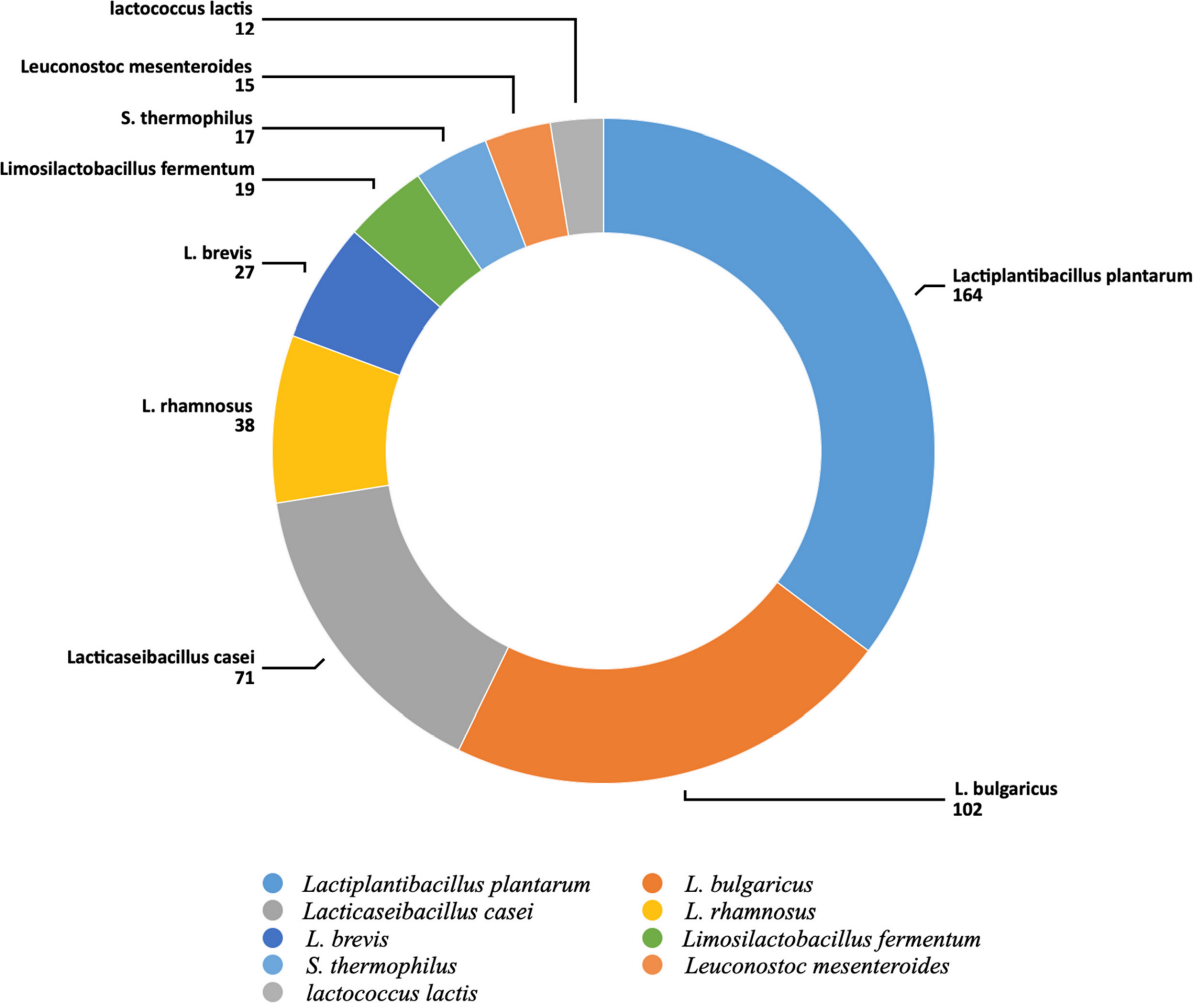
Number of confirmed *Lactobacillus* bacteria in samples

A number of colonies collected from each type of cheese and the number of confirmed LAB in samples are shown in Figures 2 and 3, respectively. Biochemical and physiological analyses confirmed 465 of the 489 samples as LAB consisting 164 *Lactip*. *plantarum* (35.26%), 102 *Lactobacillus bulgaricus* (21.93%), 71 *Lactic*. *casei* (15.26%), 38 *Lactobacillus rhamnosus* (8.17%), 27 *Lactobacillus brevis* (5.80%), 19 *Li*. *fermentum* (4.08%), 17 *Streptococcus thermophilus* (3.65%), 15 *Leuconostoc mesenteroides* (3.22%), and 12 *Lactoc*. *lactis*(2.58%). Most *Lactip. plantarum* were found in Liqvan cheese, followed by Kordi cheese. Of all the biochemically confirmed isolates, 15 random samples from each species group were chosen for further analysis (bile resistance, resistance to simulated gastrointestinal juices, antibiotic resistance test, and antibacterial activity).

Badis, Guetarni, Moussa‐Boudjemaa, et al. (2004) examined 158 raw goat milk samples from Algeria, and they isolated 32 strains of *Lactoc*. *lactis*, 19 strains of *L. bulgaricus*, 16 strains of *Lactococcus helveticus*, and 14 strains of *Lactip. plantarum*. Milani et al. (2017) isolated LAB from Kurdish cheese during 60 days of ripening. They reported that the predominant LAB were *Lactip. plantarum* which was consistent with the present study. This was also confirmed by Navidghasemizad et al. (2009), who demonstrated that *Lactip*. *plantarum* was predominant among LAB in Lighvan cheese. *Lactiplantibacillus plantarum* showed most of the criteria for being a probiotic strain as many other studies. Its high adaptable properties are due to the enzymatic ability, carbohydrate metabolism, and owning a large genome (Morelli et al., 2004). According to the study of Milani et al. (2017), the microbial community of LAB was dynamic during ripening, and at the final phase, *Lactip*. *plantarum* and *Lactobacillus paracasei* were the predominant LAB. This dynamic can be affected by factors such as temperature, season, and the ability of bacteria to utilize nutrient resources which may lead to alteration of the predominant bacteria.

### 3.2 Bile resistance

Ingested probiotic bacteria must survive the harsh conditions of the gastrointestinal tract and reach the large intestine to exert their beneficial effects. Herein, bacteria must push through several obstacles, such as bile salts. Therefore, in this study, the resistance of bacteria to several bile concentrations (0.3%, 0.5%, 1%, and 2%) were evaluated. The results are represented in Table 1. The most durable bacteria were *Lactip*. *plantarum* strains (MT1–MT6), which survived in 0.3% bile concentration. *Lactiplantibacillus plantarum* was the only bacteria that survived 0.5% bile salt for 2 h, making it the most resistant bacteria to bile in this study. The results also showed that 2% bile could eliminate significant strains even in 30 min, however, 0.3% bile concentration is more similar to the gastrointestinal conditions (Gharbi et al., 2019) than other concentrations used in this study.

**TABLE 1:** Resistance of 15 identified lactic acid bacteria isolated from traditional cheeses to 0.3%, 0.5%, 1%, and 2% bile concentrations

	Hour	Bacterial growth^a^															
		30 min				1 h				1.5 h				2 h			
	%	0.3	0.5	1	2	0.3	0.5	1	2	0.3	0.5	1	2	0.3	0.5	1	2
*Lactiplantibacillus plantarum*	80	+	+	+	+	+	+	+	−	+	+	+	−	+	+	−	−
*Lactobacillus bulgaricus*	40	+	+	+	−	+	−	−	−	+	−	−	−	+	−	−	−
*Lacticaseibacillus casei*	13.3	+	+	−	−	−	−	−	−	−	−	−	−	−	−	−	−
*Lactobacillus rhamnosus*	33.3	+	+	+	−	+	−	−	−	+	−	−	−	−	−	−	−
*Lactobacillus brevis*	40	+	+	+	+	+	+	−	−	−	−	−	−	−	−	−	−
*Limosilactobacillus fermentum*	46.6	+	+	+	−	+	+	−	−	+	+	−	−	−	−	−	−
*Streptococcus thermophilus*	53.3	+	+	+	+	+	+	+	−	+	−	−	−	−	−	−	−
*Leuconostoc mesenteroides*	26.6	+	+	+	−	+	−	−	−	−	−	−	−	−	−	−	−
*Lactococcus lactis*	33.3	+	+	−	−	+	+	−	−	−	−	−	−	−	−	−	−

^a^ Bacterial growth: +; No growth: −.

Monteagudo‐Mera et al. (2012) investigated 11 different strains of LAB from various origins such as cow and eve milk, cheese, American Type Culture Collection, and human feces for potential probiotic properties. They reported excellent bile resistance for all LAB strains in their study against 0.1%, 0.2%, and 0.4% bile concentrations even after 4 h. Moreover, another study showed that all LAB strains collected from various dairy products were resistant to 0.3% bile for 4 h (Maragkoudakis et al., 2006). Similar results were reported by Plessas et al. (2017).

### 3.3 Determination of resistance to simulated gastrointestinal juices

Other obstacles for probiotic bacteria are gastrointestinal juices such as pepsin and trypsin. The tolerance of LAB strains to gastric pepsin and trypsin is presented in Table 2. Within the first hour of treatment with pepsin at pH 2.5, all bacterial strains showed resistance, but *Lactip*. *plantarum* exhibited the best adaptation followed by *Li*. *fermentum*, while *Lactic*. *casei* was the weakest strain. After 4 h of treatment in pH 2.5, *Lactip*. *plantarum* strains (MT1–MT6) were also the most resistant bacteria with six survived strains. On the contrary, all *Lactic*. *casei* strains were eliminated, indicating the weakest bacterial strain in this test. In addition, at pH 3 and 4, all strains showed viability after 4 h, and *Lactip*. *plantarum* strains (MT1–MT6) were also the most durable strains in these conditions. Subsequently, after this test, bacterial strains were treated with trypsin adjusted to pH 8.0. Although, *Lactip*. *plantarum* strains were more resistant than other strains, all strains were viable after 12 h. These findings were in accordance with the data obtained by Kim et al. (2021).

**TABLE 2:** Pepsin and trypsin resistance of 15 identified lactic acid bacteria isolated from traditional cheeses in different pH and duration (survival percentage)

	Hour	Pepsin												Trypsin					
		pH = 2.5				pH = 3				pH = 4				pH = 8					
		1	2	3	4	1	2	3	4	1	2	3	4	1	2	3	4	8	12
*Lactiplantibacillus plantarum*		100	100	86.6	40	100	100	86.6	40	100	100	93.3	80	86.6	86.6	86.6	86.6	80	60
*Lactobacillus bulgaricus*		86.6	66.6	53.3	13.3	86.6	66.6	60	26.6	86.6	86.6	80	66.6	80	80	80	66.6	60	46.6
*Lacticaseibacillus casei*		53.3	46.6	40	0	53.3	46.6	40	13.3	53.3	53.3	46.6	26.6	40	40	33.3	33.3	26.6	20
*Lactobacillus rhamnosus*		80	66.6	53.3	20	80	73.3	66.6	40	80	80	66.6	33.3	66.6	66.6	66.6	53.3	46.6	46.6
*Lactobacillus brevis*		86.6	73.3	60	13.3	86.6	73.3	66.6	40	86.6	73.3	66.6	40	80	80	80	73.3	66.6	53.3
*Limosilactobacillus fermentum*		93.3	80	40	6.6	93.3	86.6	66.6	40	93.3	80	66.6	60	86.6	86.6	80	73.3	66.6	53.3
*Streptococcus thermophilus*		80	66.6	40	13.3	80	73.3	53.3	40	80	80	66.6	60	73.3	66.6	66.6	60	46.6	46.6
*Leuconostoc mesenteroides*		86.6	80	46.6	20	86.6	80	60	40	86.6	80	66.6	53.3	80	80	66.6	60	53.3	46.6
*Lactococcus lactic*		91.6	75	33.3	8	91.6	83.3	66.6	41.6	91.6	83.3	75	58.3	83.3	75	75	66.6	58.3	58.3

Zago et al. (2011) reported that 98 *Lactip. plantarum*, isolated from cheese, were adapted to pH 2.5 within the first hour. In another study on 11 different strains of LAB at pH 2, all strains were eliminated within 45 min of simulated gastric juice treatment, and only *Lactoc*. *lactic* survived (Monteagudo‐Mera et al., 2012). They also reported that when simulated gastric juice treatment was performed at pH 2.5, all LAB strains exhibited adaptation during the first 45 min. In the latter condition, *L. rhamnosus* and *Lactoc*. *lactic* showed viability even after 90 and 180 min, respectively. Another study showed that LAB strains were excellent tolerant of simulated intestinal juices at pH 8.0 (Guo et al., 2009). Escobar‐Ramírez et al. (2020) isolated LAB from a traditional beverage known as pineapple tepache. They reported that *Lactip*. *plantarum* was the best microorganism in low pH conditions. Several factors were suggested for the resistance of *Lactip*. *plantarum* to tolerate the acidic environment. Hamon et al. (2014) stated that dTDP‐glucose 4,6‐dehydratase, 3‐oxoacyl‐synthase II, and dTDP‐4‐dehydrorhamnose 3,5‐epimerase play an essential role in the biogenesis of cell envelope and grant the *Lactip*. *plantarum* durability in acidic conditions. In addition, high amounts of enzymes and amino acids and cell membrane integrity were proposed as influential factors to prevent bacterial death from acid stress (Guo et al., 2017).

### 3.4 Antibiotic resistance test

Antibiotic resistance is one of the most important criteria for the selection of probiotic strains. Transferable genetic materials such as antibiotic resistance genes can be spread to pathogenic bacteria. The result of the antibiotic resistance test is depicted in Table 3. Interestingly, *Li*. *fermentum* was exhibited as the highest resistance bacteria and survived more than other strains against erythromycin, streptomycin, tetracycline, cefotaxime, kanamycin, meropenem, and ceftazidime. *Lactiplantibacillus plantarum* and *L. brevis* were the most resistant bacteria to amoxicillin, while *Lactoc*. *lactic* was the most resistant bacteria to ampicillin. In the case of vancomycin, both *Li*. *fermentum* and *Lactoc*. *lactic* were more resistant. *Lactococcus lactic* also showed the most resistance to chloramphenicol. Herein, *L. bulgaricus* showed the lowest susceptibility to nalidixic acid and gentamycin. Moreover, although *Li*. *fermentum* and *L. rhamnosus* exhibited relative resistance to ceftazidime, other species showed higher susceptibility to this antibiotic. Selected probiotic strains (MT1–MT6) also showed resistance to amoxicillin, ampicillin, chloramphenicol, and erythromycin.

**TABLE 3:** Antibiotic resistance of 15 identified lactic acid bacteria isolated from traditional cheeses (survival percentage)

	*Lactiplantibacillus plantarum*	*Lactobacillus bulgaricus*	*Lacticaseibacillus casei*	*Lactobacillus rhamnosus*	*Lactobacillus brevis*	*Limosilactobacillus fermentum*	*Streptococcus thermophilus*	*Leuconostoc mesenteroides*	*Lactococcus lactic*
Amoxycillin	80	66.6	73.3	66.6	80	60	53.3	66.6	58.3
Ampicillin	73.3	40	53.3	60	53.3	60	40	60	100
Chloramphenicol	53.3	13.3	40	53.3	60	53.3	60	13.3	91.6
Erythromycin	53.3	53.3	33.3	26.6	33.3	80	33.3	33.3	50
Streptomycin	40	26.6	33.3	40	40	66.6	26.6	40	50
Tetracycline	53.3	40	40	53.3	26.6	73.3	40	53.3	66.6
Vancomycin	20	33.3	46.6	60	33.3	80	33.3	66.6	100
Cefotaxime	13.3	26.6	33.3	33.3	40	80	53.3	80	83.3
Kanamycin	20	40	60	40	13.3	86.6	33.3	73.3	33.3
Meropenem	40	53.3	26.6	53.3	40	93.3	53.3	33.3	41.6
Nalidixic acid	33.3	60	53.3	26.6	20	53.3	46.6	13.3	50
Gentamycin	40	53.3	40	40	33.3	40	40	40	41.6
Ceftazidime	13.3	26.6	33.3	46.6	26.6	53.3	13.3	13.3	16.6

Islam et al. (2021) isolated LAB strains from goat milk and evaluated their characteristics. They found that 30 µg tetracycline and neomycin could inhibit all LAB strains, while roughly all of them were resistant to vancomycin, penicillin, chloramphenicol, and ampicillin. Islam et al. (2021) also stated that only *Li*. *fermentum* and *Lactip*. *plantarum* were resistant to gentamycin and streptomycin. In another study, 10 different types of traditional cheeses in Brazil were assessed for the isolation of LAB strains (Margalho et al., 2020). The results showed that streptomycin could not prevent the growth of LAB strains, while tetracycline was the most potent antibiotic against them, followed by erythromycin, ceftazidime, and penicillin. Other studies showed that *St. thermophilus*, *Leu. mesenteroides* (Ammor et al., 2007), and several LAB strains (Ouwehand et al., 2016) were resistant to streptomycin, gentamycin, and kanamycin. Hashemi et al. (2014) reported resistance of *Lactip*. *plantarum* strains isolated from Kurdish cheese to vancomycin and streptomycin.

Antimicrobial resistance of probiotic bacteria could be intrinsic, which might be ascribed to chromosomal mutation or acquiring plasmid from other bacteria. It should be noticed that antibiotic‐resistant probiotics with adopted mobile genetic material should not be added to feed and food to prevent the spread of antibiotic resistance.

### 3.5 Antimicrobial activity of probiotic strains

Probiotic strains isolated from traditional cheeses were screened for antimicrobial activity against five different microorganisms. The results are shown in Table 4. *Limosilactobacillus fermentum* showed the highest antimicrobial activity against *Lis*. *monocytogenes*. The highest antimicrobial activity for *S. aureus* was recorded by *L. rhamnosus*, *Li*. *fermentum*, and *St. thermophilus*. *Lactobacillus brevis* and *Leu. mesenteroides* exhibited the highest growth inhibition for *Sal*. *typhimurium*. In the case of *P. aeruginosa* and *E. coli*, *L. rhamnosus* displayed the highest activity. The lowest antimicrobial activity was demonstrated for *Lactic*. *casei* compared to other probiotics. Extracellular secretions of LAB contain various compounds which act as growth inhibitors for other bacteria, such as pathogenic bacteria. Organic acids, bacteriocins, and H_2_O_2_ are of the main inhibitors of pathogenic bacteria, which can be found in extracellular secretions (Dasari et al., 2014).

**TABLE 4:** Antimicrobial activity of 15 identified lactic acid bacteria isolated from traditional cheeses against five different pathogenic bacteria (sensitivity percentage)

	*Listeria monocytogenes*	*Staphylococcus aureus*	*Salmonella typhimurium*	*Pseudomonas aeruginosa*	*Escherichia coli*
*Lactiplantibacillus plantarum*	86.6	80	93.3	73.3	66.6
*Lactobacillus bulgaricus*	80	66.6	73.3	86.6	66.6
*Lacticaseibacillus casei*	53.3	40	26.6	33.3	66.6
*Lactobacillus rhamnosus*	80	93.3	86.6	100	93.3
*Lactobacillus brevis*	80	86.6	100	66.6	86.6
*Limosilactobacillus fermentum*	100	93.3	86.6	93.3	86.6
*Streptococcus thermophilus*	80	93.3	66.6	66.6	60
*Leuconostoc mesenteroides*	80	86.6	100	80	86.6
*Lactococcus lactic*	100	100	83.3	75	66.6

Cui et al. (2018) isolated LAB from traditional cheese from China and reported broad antimicrobial activity of 12 of 37 against Gram‐positive and Gram‐negative bacteria such as enteropathogenic bacteria. They neutralized the pH of cell‐free supernatant of *Lactip*. *plantarum* and *Lactoc*. *helveticus* and observed complete elimination of the inhibition zone for these probiotic strains. They concluded that the antibacterial activity of *Lactip*. *plantarum* and *Lactoc*. *helveticus* strains should be ascribed to the production of organic acids. Cui et al. also reported that *Pediococcus acidilactici*, *Enterococcus faecium*, and two other *Lactip*. *plantarum* strains produce hydrogen peroxide because their antibacterial activity was removed after neutralization of cell‐free supernatant by catalase. In the study of Cui et al. (2018), other bacterial cell‐free supernatants were treated with proteases such as proteinase k and trypsin, after neutralization of pH and addition of catalase. This experiment was also eliminated by the antimicrobial activity of remaining isolated bacteria from Chinese traditional cheese, indicating the probability of producing bacteriocins by these remaining bacteria. Cui et al. study also showed that LAB may exhibit their antibacterial activity by the production of three mentioned compounds.

Lactic acid bacteria were isolated from different dairy products, and results showed that *Pe. acidilactici* and *Lactic*. *casei* were the most potent bacterial strains against *E. coli* and *S. aureus* (García‐Cano et al., 2019). *Lactoc*. *lactis* strains isolated from dairy milk in Algeria were able to inhibit the growth of *Listeria innocua* (Mezaini et al., 2009). LAB isolated with other origins also exhibited antimicrobial activity. Luo et al. (2015) showed that LAB isolated from a Chinese traditional fermented vegetable (pao cai) inhibited the growth of *Salmonella*.

In this study, screening all performed tests were reviewed, and finally seven promising isolates that had shown better results like resistance more than 1.5 h against bile salt concentration and better resistance to simulated gastrointestinal juices (pH = 2.5) for 4 h and 12 h were subjected for *16S rRNA* sequencing. Results demonstrated agreement between the phenotypic and genotypic methods in recognizing isolates (MT1–MT6) except for MT3, which showed the most similarity to *Lactoc. lactis* (Figure 1).

## 4 CONCLUSION

In this study, we investigated 10 traditional Iranian cheeses to isolate new probiotic strains. We also tested their adaptation to gut conditions, antimicrobial activity, resistance to pathogenic bacteria, and phylogenetic analysis. The data showed that among the different species of LAB isolated in this study, *Lactip. plantarum* was the most isolated species, and six isolates had the significant criteria for being a probiotic strain than other isolates indicating the most adaptable properties of this species. Also, this study showed the excellent potential of local cheeses of this region of Iran in terms of having probiotic strains, which can be considered as suitable biopreservatives and can be used as specific starter cultures for the production of functional cheeses. It should be taken into consideration that analysis of technological properties of isolated probiotics was one of the limitations of our study, and should be carried out in further studies. Isolated probiotic bacteria can be added to different foods and their desirable effects, such as antimicrobial activity, should be tested.

## CONFLICT OF INTEREST

The authors declare no conflict of interest.

## Data Availability

Data available on request from the authors.
